# Aversive Training of Honey Bees in an Automated Y-Maze

**DOI:** 10.3389/fphys.2019.00678

**Published:** 2019-06-04

**Authors:** Morgane Nouvian, C. Giovanni Galizia

**Affiliations:** ^1^Department of Biology, University of Konstanz, Konstanz, Germany; ^2^Centre for the Advanced Study of Collective Behaviour, University of Konstanz, Konstanz, Germany

**Keywords:** honey bees, aversive learning, automation, Y-maze, bimodal

## Abstract

Honeybees have remarkable learning abilities given their small brains, and have thus been established as a powerful model organism for the study of learning and memory. Most of our current knowledge is based on appetitive paradigms, in which a previously neutral stimulus (e.g., a visual, olfactory, or tactile stimulus) is paired with a reward. Here, we present a novel apparatus, the yAPIS, for aversive training of walking honey bees. This system consists in three arms of equal length and at 120° from each other. Within each arm, colored lights (λ = 375, 465 or 520 nm) or odors (here limonene or linalool) can be delivered to provide conditioned stimuli (CS). A metal grid placed on the floor and roof delivers the punishment in the form of mild electric shocks (unconditioned stimulus, US). Our training protocol followed a fully classical procedure, in which the bee was exposed sequentially to a CS paired with shocks (CS+) and to another CS not punished (CS-). Learning performance was measured during a second phase, which took advantage of the Y-shape of the apparatus and of real-time tracking to present the bee with a choice situation, e.g., between the CS+ and the CS-. Bees reliably chose the CS- over the CS+ after only a few training trials with either colors or odors, and retained this memory for at least a day, except for the shorter wavelength (λ = 375 nm) that produced mixed results. This behavior was largely the result of the bees avoiding the CS+, as no evidence was found for attraction to the CS-. Interestingly, trained bees initially placed in the CS+ spontaneously escaped to a CS- arm if given the opportunity, even though they could never do so during the training. Finally, honey bees trained with compound stimuli (color + odor) later avoided either components of the CS+. Thus, the yAPIS is a fast, versatile and high-throughput way to train honey bees in aversive paradigms. It also opens the door for controlled laboratory experiments investigating bimodal integration and learning, a field that remains in its infancy.

## Introduction

Karl von Frisch performed the earliest conditioning experiments on honey bees, simultaneously demonstrating that they can see colors and that they can learn color-reward associations (von Frisch, 1914). Since then, numerous studies have used learning paradigms to gain insights into how honey bees perceive and understand the world (Menzel, 2001; Srinivasan, 2010; Sandoz, 2011; Avargues-Weber and Giurfa, 2014). The vast majority of this work focused on appetitive learning, in which one stimulus (the conditioned stimulus, CS) is paired with a reward – usually a droplet of sugary water (the unconditioned stimulus, US). In outdoor settings, the presence of a reward can lure honey bees to willingly participate in the experiment. They will then shuttle back and forth from the hive to the experimental set-up, thus inscribing the learning process in the naturalistic context of foraging. Typically, at the experimental station the bees are given the choice between two alternatives, only one of which being rewarded, that they can examine at leisure before making a choice. This approach has been extensively used to probe the limits of honey bee cognition, especially in visual tasks (Giurfa et al., 2001; Hempel de Ibarra et al., 2014; Howard et al., 2018) but not exclusively (Srinivasan et al., 1998; Ravi et al., 2016). Among the major drawbacks of using free-flying bees, however, is the difficulty to gain insights into the underlying neural circuits, either through pharmacology or electrophysiology. Such a mechanistic understanding necessitates well-controlled lab-based experiments. The most famous procedure that fits this criterion is the conditioning of the proboscis extension response (PER), which has been instrumental in establishing the honey bee as a model system for learning and memory. Harnessed bees are presented repeatedly with the CS while their antennae is stimulated with a droplet of sugar, thus releasing a reflexive extension of the proboscis to lick the sugar. After a few trials the CS itself triggers extension of the proboscis, thus indicating Pavlovian conditioning (Giurfa and Sandoz, 2012). While olfactory PER produces high learning rates and a robust memory, adapting this protocol to visual tasks has proven challenging (Avargues-Weber and Mota, 2016).

Comparatively, aversive paradigms are still few and rarely used, although recent efforts have been made to close this gap. These includes conditioning of the sting extension reflex, in which the bees are harnessed and the CS (an odor) is paired with either electric shocks or heat (Vergoz et al., 2007; Tedjakumala and Giurfa, 2013; Junca et al., 2014). More recently, place avoidance assays have been developed (Agarwal et al., 2011; Dinges et al., 2013; Kirkerud et al., 2017). In these assays the walking bee receives shocks when she enters the half of the arena marked by a specific color, and thus learns to escape to the other side, marked by a different color. Nonetheless, a major issue of these assays was that the bee always started within one of the stimuli, and was thus never in the position of evaluating both alternatives before making a choice. In addition, how the bee was learning (whether it was operant, classical or a mix of both) was never clear. Here, we present a new apparatus, the Y-APIS, that solve these issues. The shape of the arena was modified from a linear chamber to a Y-maze, and each arm was fitted with colored lights and an odor delivery system. Real-time tracking inside the apparatus allowed for any stimulus (CS or US) to be delivered relative to the position of the bee, so that the bee could be offered a real choice between two alternatives. The Y-maze (or T-maze) is a simple but powerful tool for the study of animal behavior, and it has been used to assess decision-making and sensory perception in a wide range of species, from slime molds (Reid et al., 2012) to rodents (Flexner et al., 1963), birds (Bonadonna and Sanz-Aguilar, 2012) and fish (Cognato et al., 2012), passing by insects (Giurfa et al., 2001).

To validate our approach we trained honey bees in a simple differential conditioning, in which the CS+ was paired with shocks but not the CS-. We show that honey bees successfully solved the task after only a few training trials both in the visual and in the olfactory modality, with an exception when the CS+ was within the UV range. Further experiments revealed that they did so by avoiding the CS+ rather than by increased attraction to the CS- (i.e., there was no safety learning). As a final proof of concept, we trained honey bees to a bimodal task, in which a color and an odor were paired with the shocks simultaneously. Both sensory modalities were equally efficient in triggering the avoidance response, thus suggesting that the Y-APIS could be a powerful tool for future investigations focusing on sensory integration and learning across modalities.

## Materials and Methods

### Honey Bees

During summer, the bees were caught from outdoor colonies as they flew away and are thus most likely foragers. The bee colonies (*Apis mellifera*) were housed on the roof of the University of Konstanz, Germany. During winter, honey bees were taken from caged colonies kept indoor under a 12/12 h light-dark cycle (including UV lights) and provided with pollen, liquid bee food (APIFONDA^®^) and water to forage on. They were caught when flying in the meshed cage, outside of the hive. All bees were introduced in the apparatus immediately after being caught. Table 1 recapitulates the bees participating in the different experiments. Bees from experiments 1 and 2 were pooled to analyze the general behavior of bees inside the apparatus. To allow more direct comparisons, data from a single bee sometimes contribute to more than one figure: Figure 6B, 8B are subsets of the dataset fully presented in Figure 7, and Figure 5B is a subset of the data fully presented in Figure 8A.

**Table 1:** Overview of experiments.

Experiment	CS	Season	Sample size	Figures
Calibration	Colors	Winter	3 groups × 18 bees	1E
1	Odors	Winter	4 Nt × 2 symmetrical trainings × 20 bees	3B, 4, 5A
1	Colors	Winter	3 color pairs × 4 Nt × 2 symmetrical trainings × 20 bees	3B, 4, 6A
2	Odors	Summer	(2 symmetrical trainings + 1 control) × 48 bees	3B,C, 4A–D, 5B, 8A
2	Colors	Summer	3 color pairs × (2 symmetrical trainings + 1 control) × 48 bees	3B,C, 4A–D, 6B, 7, 8B
3	Odors	Summer	(2 symmetrical trainings + 2 controls) × 48 bees	9A
3	Colors	Summer	(2 symmetrical trainings + 2 controls) × 48 bees	9B
3	Bimodal	Summer	(2 symmetrical trainings + 2 controls) × 48 bees	9C


### Training Apparatus: Y-APIS

**FIGURE 1:**
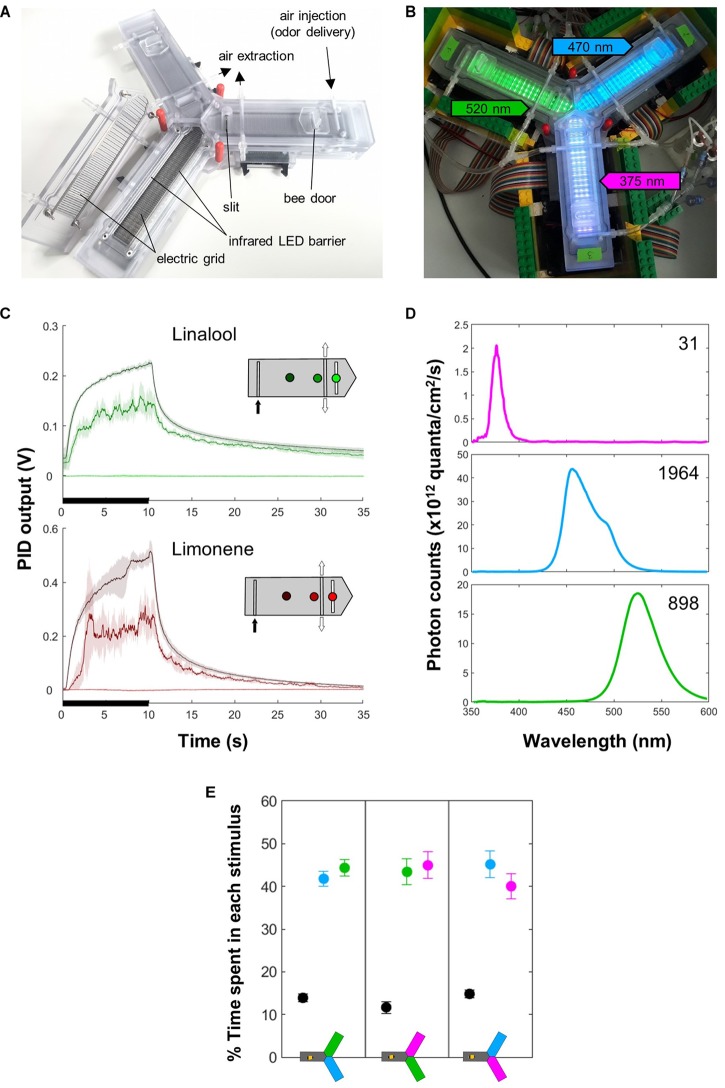
Apparatus. **(A)** The apparatus. Each arm is equipped with an entry door, an infrared LED barrier that records the position of the bee, and two electric grids (on the floor and ceiling) that serve for delivery of mild electric shocks (US). A constant air flow (in which odors can be injected) runs from the distal part of the system and is evacuated centrally through an active vacuum and a passive slit. The LEDs used as CS are situated below the floor. **(B)** The apparatus in place, with the different lights on: green (520 nm) in arm 1, blue (470 nm) in arm 2, UV (375 nm) in arm 3 (the intensities do not match what was used for experiments). **(C)** PID measurements show that odor delivery (black bars) is well defined spatially and temporally. Each curve corresponds to a position within the arm, as indicated on the inset. Mean ± SD of five consecutive measurements spaced by 30 s. The insets indicate the different points where the measures were taken inside the arm. The odor was injected at the distal end (black arrow) and evacuated centrally by the vacuum (white arrows). **(D)** Light spectrum of the different LEDs, measured inside the apparatus at the intensities used during the experiments. The number is the upper right corner indicates the total photon count (×10^12^). **(E)** The light intensities were chosen so that naïve bees did not exhibit preferences between any two lights in a choice test. All lights elicited strong positive phototaxis. *n* = 18 bees × 3 groups.

Honey bees were trained individually in an automated Y-maze custom-build at the University of Konstanz (Figure 1A,B). This apparatus was a modification of a previous linear chamber (Kirkerud et al., 2013, 2017). It is made of three arms (inner dimensions: 14 cm long, 2 cm wide and 0.55 cm high), at 120° from each other. Individual bees were inserted via one of the upper doors and allowed to walk freely in all three arms. The position of the bee in each arm was monitored using an array of 26 infrared photocells. The US consisted of a train (2 Hz) of mild electric shocks (10 V for 200 ms) delivered by the electric grid placed on the floor and ceiling of each arm. The CS consisted in odors delivered into the air stream at the distal end of each arm, or light delivered along the entire length of an arm.

#### Odors

A constant clean air flow (300 mL/min) ran from the distal end of each arm to the central area, where it was evacuated by active vacuuming and a passive slit (Figure 1A,B). Smaller air flows (100 mL/min) joined the main one just before its entry into each arm. By default, each flow ran through a clean vial, and for odor delivery its path was switched to a similar vial containing 0.5 mL of the pure odorant, using solenoid valves (Bürkert 6724, FFKM/PEEK). The odorants were R-(+)-limonene and linalool (Sigma-Aldrich, >94% – CAS:5989-27-5 and >97% – CAS: 78-70-6, respectively). Honey bees can learn and discriminate these odors (Kirkerud et al., 2013). Using a photo-ionization device (PID, Aurora Scientific Inc., 200A) we confirmed that the odor was well defined spatially and temporally (Figure 1C).

#### Colors

Bees have trichromatic vision, ranging from the human green to UV. Monochromatic LEDs were situated underneath the transparent floor of the apparatus in alternating rows (Figure 1B). Throughout this study, we refer to them in human color space, i.e., green (λ = 520 nm, 3.5 mW LED, Kingbright KP-1608VGC-A), blue (λ = 465 nm, 27 mW LED, Kingbright KP-1608VBC-D) and ultra-violet (UV; λ = 375 nm, 9.9 mW LED, Nichia NSSU100DT). Green (520 nm) is mainly perceived by the long-wavelength receptor alone (in human trichromatic vision, activating L only yields red). Blue (465 nm) is perceived by the bees’ long- and middle-wavelength photoreceptor (in humans, L and M activation yields yellow). The UV stimulus is perceived by both middle- and short-wavelength photoreceptors (in humans, activating M and S photoreceptors yields a bright blue). Honey bees have very good color discrimination within these regions of the visual spectrum, and should thus be able to easily identify these different stimuli (Avargues-Weber and Giurfa, 2014).

In preliminary experiments, we found that forager bees were strongly phototactic, and the phototactic strength depended on the wavelength. Therefore, we calibrated light intensities to equalize their preference in a two-choice test (Figure 1E). This corresponded to 64, 44, and 24% of the maximum intensity for 520, 465 and 375 nm, respectively. The exact spectra and their intensity with these settings, as seen from inside the apparatus, are presented in Figure 1D.

### Training Procedures

**FIGURE 2:**
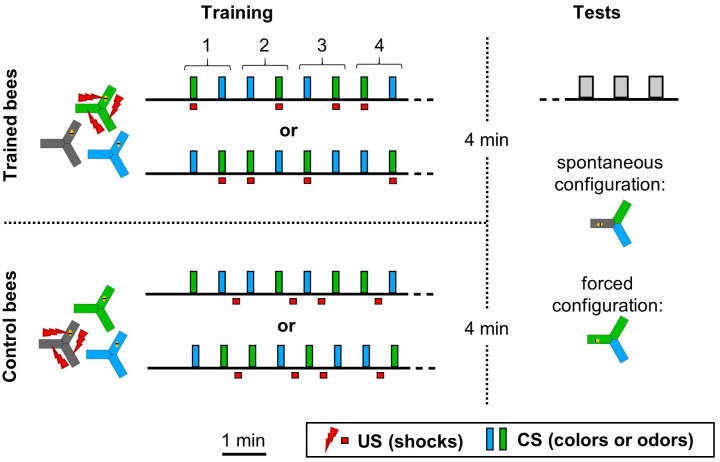
Experimental design. During the training phase the CS and/or US were presented throughout the entire apparatus for 10 s. Honeybees were trained with one of two training sequences, such that half of the bees were trained with sequence 1 and the other half with sequence 2. Control bees were subjected to the same CS sequences, but with the shocks unpaired from both CS. In this example, the bees had four training trials with each CS. Performance could be tested with two CS configurations, depending on the experiment. In the “spontaneous” choice, the CS were placed so that the bee started in the dark arm and was confronted with a choice between the two other arms. In the case of colors, the bee was naturally willing to make a choice due to positive phototaxis. In the case of odors, these two choice arms were made attractive by a dim blue illumination. In the “forced” choice, two arms contained the CS+, including the one where the bee started. The last arm always contained the CS–. In this configuration, the odor choice was tested in the dark. No shocks were ever given during the tests, which lasted 20 s.

Acquisition trials (Figure 2) followed a classical differential conditioning protocol. After 1 min of adaptation to the apparatus in the dark, we exposed the bees to a variable number of training trials. Each trial consisted of 10 s exposure to the CS (light or odor throughout the apparatus) followed by 30 s of inter-trial interval (dark/no odor), then 10 s to the other CS also followed by 30 s rest. For trained animals, the US shocks were paired with the CS+ during the full 10 s of exposure. For control animals, the shocks were given also for 10 s but in the middle of the inter-trial interval (from 10 to 20 s after the end of a CS). The bees reacted to the shocks as previously described, by accelerating and hissing (Kirkerud et al., 2013; Wehmann et al., 2015). Sequences were pseudo-randomized (Figure 2). After the training phase, all bees had 4 min to rest in the dark before the start of the testing phase. Up to four yAPIS were used in parallel, and within each experimental group the bees were distributed equally on these systems. This also allowed for associated groups (e.g., blue shocked vs. green, green shocked vs. blue and the corresponding unpaired control group) to be tested in parallel whenever possible.

### Memory Testing

Each test lasted for 20 s, followed by 30 s of rest. Thanks to the real time tracking of the bee available in our apparatus, the stimuli could be presented relative to the bee position. Two stimuli configurations were used, depending on the experiment (Figure 2). In the “spontaneous” configuration, two CSs were presented relative to the bee position, such that the bee always started in the dark and faced the choice between the two stimuli. In the case of colors, the bee was naturally willing to make a choice due to positive phototaxis. In the case of odors, these two choice arms were made attractive by a dim blue illumination (2.5% of maximum intensity). In the “forced” choice, two arms contained the CS+, including where the bee started. The remaining arm always contained the CS-. An advantage of this configuration was that odors could be tested in the dark, without the need for an additional light to induce phototaxis. The right/left positions of each CS relative to the bee were alternated between tests. Whenever the protocol included different tests, their order was balanced across animals. In order to test 24 h memory, trained bees were taken out of the apparatus and held individually overnight in a dark and moist chamber, with *ad libitum* solid food (APIFONDA^®^). This protocol ensured that 98% of the bees survived.

### Data Collection

The yAPIS system collected all data onto a log file. Measurements consisted in the position of the bee along the arm, the arm the bee was in, each electric shock, the current flow during the shock, the odor delivery events and the lights on/off events.

### Data Analysis

The data was analyzed using a custom script written in Python 3.7, and statistical tests were implemented in Matlab R2018b. We defined a criterion for unhealthy or exhausted bees: bees that moved slower than 6 mm/s during the test phase were excluded, and new bees were measured instead. Learning scores are based on the percentage of time spent in each light environment. Direct comparisons of the two lights in a “spontaneous” choice test were performed using Wilcoxon signed rank tests. Mann–Whitney *U*-tests were performed to observe the change in distributions between the control group and a trained group. In all statistical tables, the uncorrected *p*-value from the test are reported as “*p*,” and false discovery rate corrected *p*-values as “FDR” (Verhoeven et al., 2005). In Figure 8, we pooled the two symmetrical training groups and compared them to a single control group by attributing the roles of CS+ and CS- to the correct stimuli. Analyses using ANOVA tests were followed by a *post hoc* Tukey’s honest significant difference (HSD) test when required. The outliers were defined as values that are more than 1.5 times the interquartile range away from 25th and 75th percentiles.

## Results

### Honey Bee Behavior Inside the yAPIS System

**FIGURE 3:**
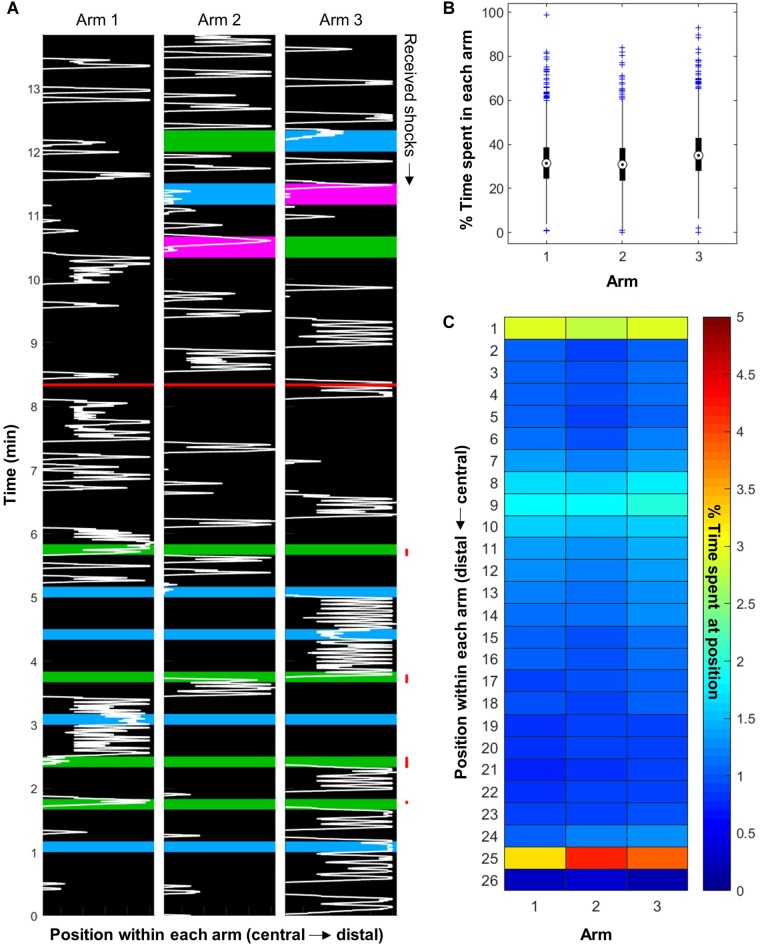
Honeybees explored the whole apparatus. **(A)** Representative example of bee movement inside the apparatus (bee trained with four training trials in the blue vs. green paradigm, with green shocked, and tested with all three color pairs). The background color represents the light inside each arm. The red line separates the training phase from the STM test. The red points on the right mark the received electric shocks. **(B)** The bees explored the whole apparatus, and had a slight preference for Arm 3 (*n* = 1216 bees). **(C)** Bees spent more time at the far end of each arm and in the central area. They also seemed to react to the presence of the vacuum, which is on top of position 9 (*n* = 576 bees).

Bees placed inside the apparatus moved constantly within, as exemplified in Figure 3A. They explored all three arms of the apparatus, with a very slight preference for Arm 3 [Figure 3B; ANOVA, *F*(2,3645) = 46.87, *p* < 0.001]. Even though the apparatus were shielded from the light inside a black fabric box, Arm 3 always faced away from the wall where it may have received more residual light than the other two arms. Looking more closely at the spatial distribution of the bees inside the apparatus revealed that they spent more time at the far end of the arms and in the central area (Figure 3C, positions 25 and 1, respectively). Furthermore, they seemed to react to the presence of the vacuum at position 9, often turning back at this point (also visible on Figure 3A).

**FIGURE 4:**
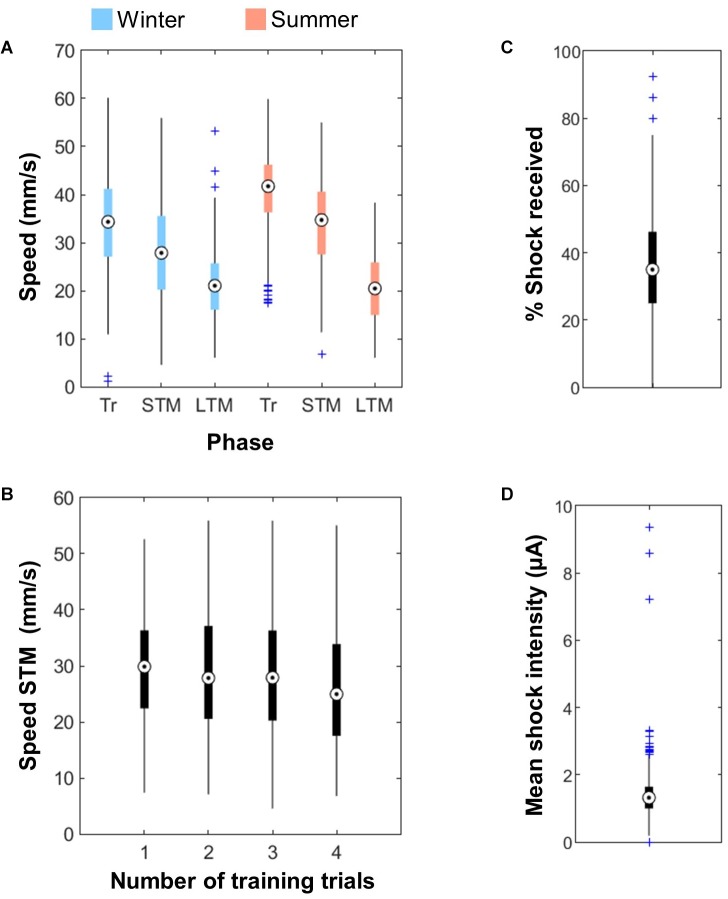
Walking speed and shocks. **(A)** Walking speed of the bees during the training phase (Tr), the STM test and the LTM test. Summer bees (*n* = 576 for Tr + STM, 288 for LTM) were slightly faster than bees reared under artificial conditions during winter (*n* = 640). The bees’ speed also depended on the phase considered. **(B)** The walking speed of the bees during the STM test was slightly reduced after 4 training trials (*n* = 640). **(C)** Around 35% of the shocks delivered were actually received by the bees (*n* = 1216 bees). **(D)** Intensity of the shocks received by the bees (mean for each individual bee, *n* = 1216).

The bees’ walking speed was 30 mm/s on average, but depended on the season: summer bees were a little bit faster than artificially reared bees (Figure 4A; ANOVA, *F*(1,3356) = 223.41, *p* < 0.001). As a result only 5% of bees were below the exclusion criteria of 6 mm/s in summer while 14% of bees were excluded in winter. In addition, the bees’ speed depended strongly on the phase considered [ANOVA, *F*(2,3356) = 807.95, *p* < 0.001; *post hoc* multiple comparisons (Tukey’s HSD): all *p* < 0.001]. Bees were fastest during the training phase, most likely as a response to the electric shocks – as already demonstrated in Kirkerud et al. (2017). Their average speed during the STM test was slower, however, the number of training trials received (and hence the duration of the training) only had a small impact on the bees’ speed during this second phase [Figure 4B; ANOVA, *F*(3,636) = 4.6, *p* = 0.0034, *post hoc* multiple comparisons (Tukey’s HSD): 1 vs. 4, *p* = 0.0055, 2 vs. 4, *p* = 0.0094]. Thus the decrease in speed between training and STM most likely resulted from the bees settling down after the shocks rather than exhaustion. Finally, bees were slowest during the LTM test, likely as a result of being contained for 24 h (even though our procedure had a very good survival rate – 98%). It could also be that they were habituated to the arena the second time.

Electric shocks delivery was quantified by measuring the current flow. A bee only received the shock when she contacted adjacent wires of the grid thus closing the circuit, which happened for 34.4 ± 13.81% of the delivered shocks (Figure 4C). Since the full US is a train of 20 shocks at 2 Hz, the bees received on average seven shocks within the 10 s of US delivery. The mean intensity of the shocks received by each bee was 1.38 ± 0.67 μA (Figure 4D). The upper outliers in these figures correspond to bees that defecated inside the apparatus (which was rare and only happened in winter), thereby creating short-circuits.

### Bees Learn to Associate Odors to Punishment

**FIGURE 5:**
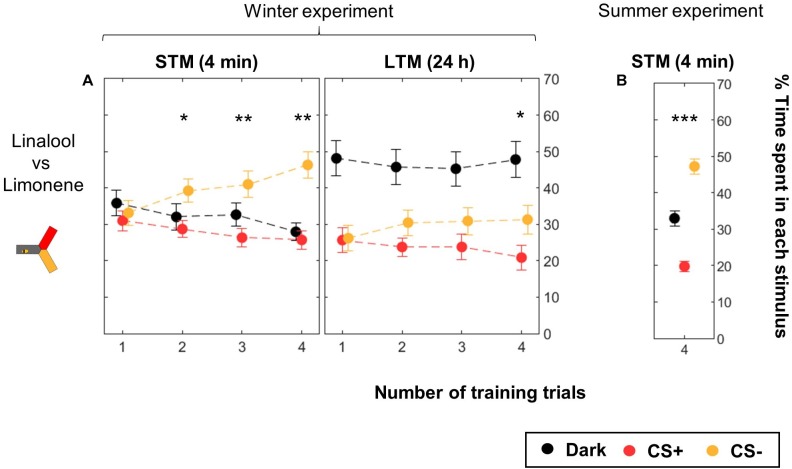
Differential conditioning of odors. During training, shocks were paired with the CS+ (red) but not with the CS- (yellow). Twenty bees were trained to one odor, and 20 bees to the other odor. The same bees were tested twice, 4 min after the end of the training and again 24 h later. Data is represented as mean ± SEM. Wilcoxon signed rank tests comparing CS+ to CS-, ^∗^*p* < 0.05, ^∗∗^*p* < 0.01, ^∗∗∗^*p* < 0.001. **(A)** Winter experiment, *n* = 160 bees (four numbers of CS presentation × 40 bees). **(B)** Summer experiment, *n* = 96 bees.

We used the yAPIS to train bees to differentiate two odors as CSs (Figure 5, Table 2, and Supplementary Figure S1). Half of the bees were trained with limonene as CS+ and linalool as CS-, while for the other half CS+ and CS- were reversed, balancing any potential bias in preference. Bees were kept in the dark during the whole training. During testing, the arm where the bee was positioned was identified, and the two odorants were delivered into the other two arms, along with a blue background illumination. Positive phototaxis induced the bees to move toward these arms, and then to make a choice between the two odorants. Each bee went through two testing sessions: one 4 min after the end of the training (short-term memory, STM), and another 24 h later (long-term memory, LTM). An avoidance of the CS+ arm against the CS- arm was observed after two or more training trials when the bees were tested shortly after the training (STM, Figure 5A). A day later, a specific memory trace was only observed in bees that received four training trials with each CS. Strikingly, bees spent more time in the dark arm during this LTM test, but the reason why (e.g., decrease of the phototactic response, avoidance of both odorants?) could not be evaluated in this dataset, and will be addressed below. These experiments were performed during winter, using a honey bee colony kept in a standardized day/light rhythm, with access to pollen and food, but not to a natural free-space environment. When we repeated the 4-trial STM experiment in summer (Figure 5B), we found similar results. This suggests that artificial rearing did not affect olfactory learning.

**Table 2:** Summary of statistical results for the olfactory conditioning (Figure 5), Wilcoxon signed-rank tests.

Odor pair	Nt	*n*	STM		LTM	
				
			*z*	*p*	*z*	*p*
Linalool vs. limonene	1	40	-0.1954	0.8451	-0.1697	0.8652
	2	40	-2.2747	**0**.**0229**^∗^	-1.3125	0.1894
	3	40	-2.6514	**0**.**008**^∗∗^	-1.1629	0.2449
	4	40	-3.0109	**0**.**0026**^∗∗^	-2.2603	**0**.**0238**^∗^


*^∗^*p* < 0.05, ^∗∗^*p* < 0.01, ^∗∗∗^*p* < 0.001.*

### Some Color Pairs Are Easier to Learn Than Others

We trained 12 groups of 40 bees, corresponding to four numbers of training trials (Nt, from 1 to 4 for each CS) across the three color pairs available in the yAPIS (blue vs. green, blue vs. UV, and green vs. UV). In each group, half of the bees were trained to one color of the pair whereas the other half was trained to the other color, so that any pre-existing preference was balanced by pooling the data. Independently of the number of training trials, bees spent overall more time in the CS- (∼50%) than in the CS+ (∼30%) during the STM test (Supplementary Figure S2). This learning was highly significant (Table 3, “All pairs”). After 24 h, the learning effect disappeared in bees that received only one training trial, indicating that only short-term learning had occurred, but was maintained in all the other groups (Table 3). However, we noted that performance differed widely between color pairs.

**Table 3:** Summary of statistical results for the visual conditioning (Figure 6), Wilcoxon signed-rank tests.

Color pair	Nt	*n*	STM		LTM	
				
			*z*	*p*	*z*	*p*
All pairs	1	120	-3.4199	**0**.**0006**^∗∗∗^	-0.9064	0.3647
	2	120	-5.0756	**<0**.**0001**^∗∗∗^	-2.128	**0**.**0333**^∗^
	3	120	-5.9321	**<0**.**0001**^∗∗∗^	-3.4446	**0**.**0006**^∗∗∗^
	4	120	-3.5752	**0**.**0003**^∗∗∗^	-2.5827	**0**.**0098**^∗∗^
Blue vs. green	1	40	-2.1101	**0**.**0349**^∗^	-0.2512	0.8017
	2	40	-4.5028	**<0**.**0001**^∗∗∗^	-2.1246	**0**.**0336**^∗^
	3	40	-3.9383	**<0**.**0001**^∗∗∗^	-2.5161	**0**.**0119**^∗^
	4	40	-4.661	**<0**.**0001**^∗∗∗^	-2.5259	**0**.**0115**^∗^
Blue vs. UV	1	40	-2.2313	**0**.**0257**^∗^	-0.7317	0.4644
	2	40	-1.3676	0.1714	-0.6838	0.4941
	3	40	-4.8006	**<0**.**0001**^∗∗∗^	-1.1963	0.2316
	4	40	-0.7675	0.4428	0.3847	0.7005
Green vs. UV	1	40	-1.4651	0.1429	-0.6183	0.5364
	2	40	-2.7555	**0**.**0059**^∗∗^	-1.0319	0.3021
	3	40	-1.0215	0.307	-2.0653	**0**.**0389**^∗^
	4	40	-0.6977	0.4853	-2.1951	**0**.**0282**^∗^


*^∗^*p* < 0.05, ^∗∗^*p* < 0.01, ^∗∗∗^*p* < 0.001.*

**FIGURE 6:**
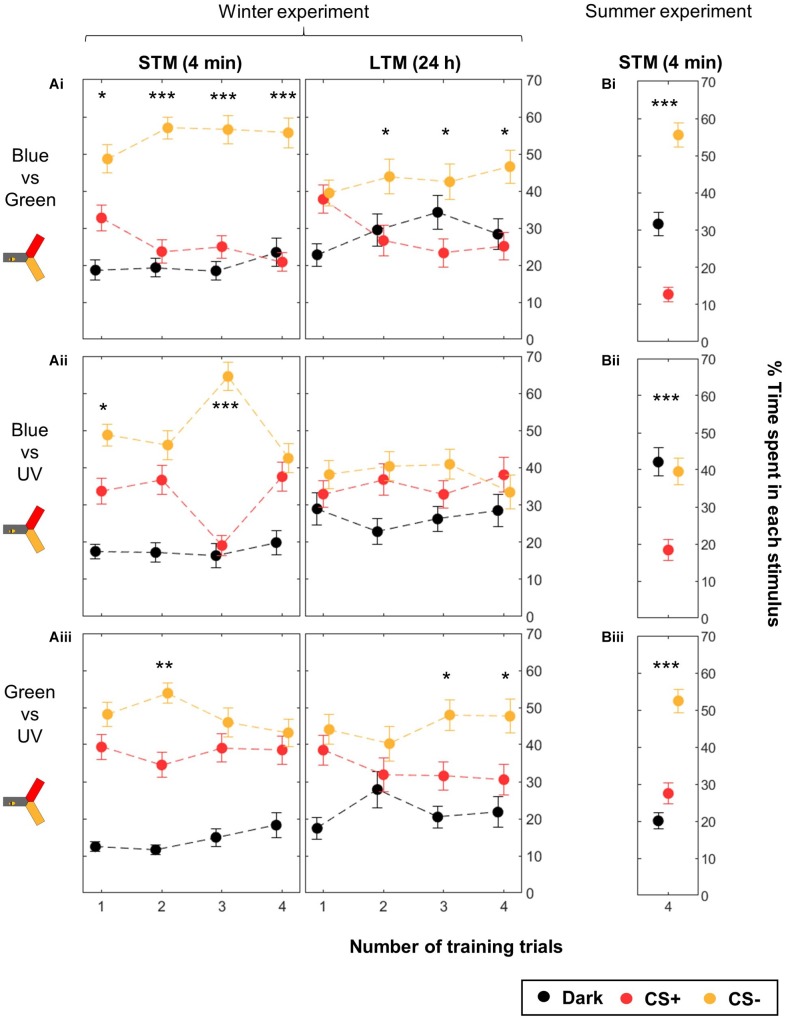
Differential conditioning of colors: learning performance depends on the color pair. During training, shocks were paired with the CS+ (red) but not with the CS– (yellow). For each color pair and each number of CS presentations, half of the bees were trained to one color, and the other half to the other color. During winter **(A)** the same bees were tested twice, 4 min after the end of the training and again 24 h later. Data is represented as mean ± SEM. Wilcoxon signed rank tests comparing CS+ to CS–, ^∗^*p* < 0.05, ^∗∗^*p* < 0.01, ^∗∗∗^*p* < 0.001. **(A)** Winter experiment, *n* = 480 bees (3 color pairs × 4 numbers of training trials × 40 bees). **(B)** Summer experiment, *n* = 144 bees (3 color pairs × 48 bees).

Therefore, we analyzed the data separating the different color groups, pooling symmetric training in order to compensate for unequal color preference (Figure 6 and Table 3). The unpooled data can be found in Supplementary Figures S3 (STM) and S4 (LTM). Here, we found that training blue vs. green produced significant STM already after 1 trial, and significant LTM after two training trials (Figure 6Ai). Training either blue or green against UV, however, did not elicit clear learning, except in few cases possibly due to random fluctuations (Figure 6Aii, Aiii; e.g., Nt = 3 STM for the blue–UV pair). Similarly, the LTM test was only significant for Nt = 3 and 4 for green vs. UV. These experiments were conducted during winter, with bees reared inside a warm cage under an artificial “sky” (including UV lights). Possibly, the difference in performance could be due to these rearing conditions. Therefore, we compared their performances to those of summer bees, by repeating the four-trial STM protocol. Summer bees performed better in all groups, and solved the task with all color pairs (Figure 6B). As for caged bees, the strongest learning scores were found for the blue vs. green condition.

**FIGURE 7:**
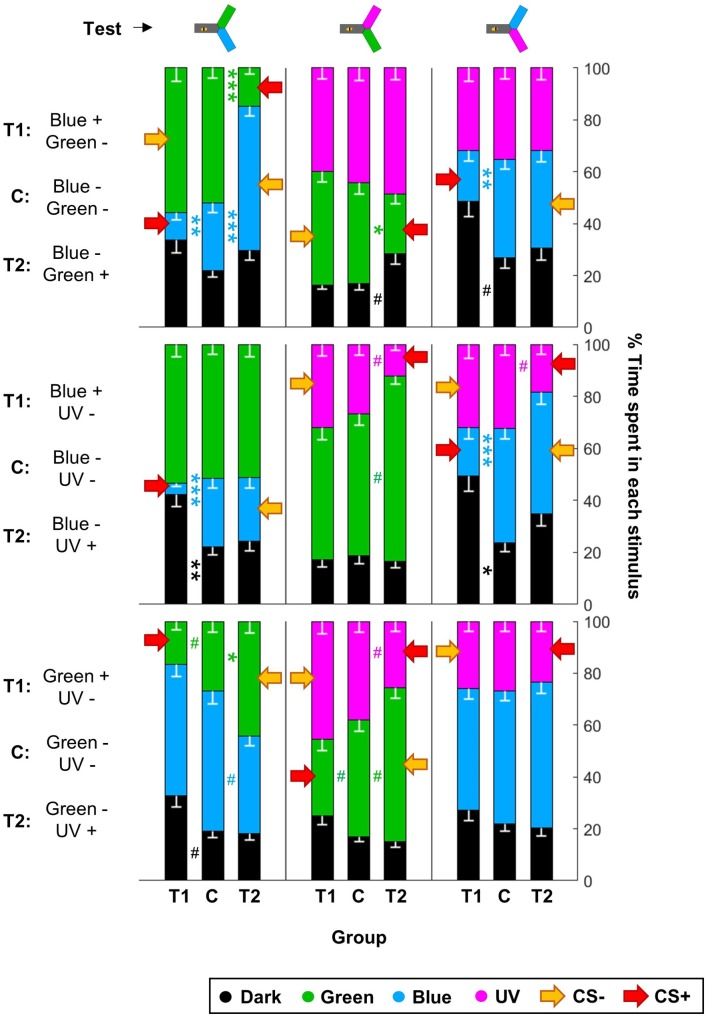
UV is resistant to aversive training. During training, shocks were paired with the CS+ but not the CS– for trained animals (T1 and T2), while control bees (C) received shocks unpaired from both CS. Each bee participated in all three tests after training. Red arrows highlight the CS+. For each group of bees, the percentages of time spent in the environments available during a given test (e.g., dark, blue, and green) are plotted and stacked into a single bar. Since we included all environments, the resulting stack always totalize 100% (the whole test duration). Data is represented as mean – SEM; *n* = 48 bees × 9 groups. Mann–Whitney *U*-tests comparing C to T1 or T2, corrected with FDR, ^∗^*p* < 0.05, ^∗∗^*p* < 0.01, ^∗∗∗^*p* < 0.001, #0.05 > *p* > corrected threshold.

Given that this analysis showed that the color pair used influenced the learning score, we analyzed whether within one pair the two colors were learned equally well or not. We trained another nine groups of 48 summer bees, including three unpaired control groups (Table 1). To get a finer understanding, we also tested the behavior of these bees when faced with all three color pairs (Figure 7 and Table 4). In this figure and the following, the data from trained bees (T) is compared to the data from the control groups (C). For each group of bees, the percentages of time spent in the environments available during a given test (e.g., dark, blue, and green) are plotted and stacked into a single bar. Since we included all environments, the resulting stack always totalize 100% (the whole test duration). In the blue/green pair, bees learned to avoid blue when punished, and to avoid green when punished. In the blue/UV pair, blue as CS+ decreased bee visits, but UV did so only marginally. In the green/UV situation, learning effects were marginal (i.e., significance disappeared with FDR correction). Thus, we concluded from this experiment that honey bees can readily attach an aversive memory to the blue and green wavelengths, but not to UV. Possibly, the phototactic attraction elicited by a light within the UV range is more resilient to aversive classical conditioning.

**Table 4:** Summary of statistical results for the second visual conditioning (Figure 7), Wilcoxon signed-rank tests, “*p* FDR” indicates the *p*-value corrected with FDR.

Group	Test	Color	*n*_t_ = *n*_c_	STM		
				
				*z*	*p*	*p* FDR
BG (T1)	B vs. G	D	48	-0.6414	0.5212	0.5864
		B	48	3.6829	**0**.**0002**^∗∗∗^	**0**.**0012**^∗∗^
		G	48	-1.0168	0.3092	0.6957
	G vs. U	D	48	-0.5019	0.6157	0.6157
		G	48	-0.814	0.4157	0.6236
		U	48	0.8134	0.416	0.5349
	B vs. U	D	48	-2.11	**0**.**0349**^∗^	**0**.**1047**
		B	48	3.7668	**0**.**0002**^∗∗∗^	**0**.**0012**^∗∗^
		U	48	0.8507	0.3949	0.7108
GB (T2)	B vs. G	D	48	-1.3007	0.1934	0.3481
		B	48	-4.9576	**<0**.**0001**^∗∗∗^	**<0**.**001**^∗∗∗^
		G	48	6.1851	**<0**.**0001**^∗∗∗^	**<0**.**001**^∗∗∗^
	G vs. U	D	48	-2.0592	**0**.**0395**^∗^	**0**.**0889**
		G	48	2.7116	**0**.**0067**^∗∗^	**0**.**0201**^∗^
		U	48	-0.613	0.5399	0.6942
	B vs. U	D	48	-0.3922	0.6949	0.7818
		B	48	0.1544	0.8773	0.8773
		U	48	0.7888	0.4302	0.6453
BU (T1)	B vs. G	D	48	-3.0354	**0**.**0024**^∗∗^	**0**.**0072**^∗∗^
		B	48	4.5918	**<0**.**0001**^∗∗∗^	**<0**.**001**^∗∗∗^
		G	48	-0.6789	0.4972	0.7458
	G vs. U	D	48	0.0916	0.9270	0.9270
		G	48	0.6343	0.5259	0.6761
		U	48	-0.8512	0.3946	0.7103
	B vs. U	D	48	-2.4309	**0**.**0151**^∗^	**0**.**0340**^∗^
		B	48	4.3551	**<0**.**0001**^∗∗∗^	**<0**.**001**^∗∗∗^
		U	48	0.2358	0.8136	0.9152
UB (T2)	B vs. G	D	48	0.2455	0.8061	0.9068
		B	48	0.3117	0.7552	0.9710
		G	48	-0.1504	0.8805	0.8805
	G vs. U	D	48	0.3261	0.7444	1.1165
		G	48	-2.5832	**0**.**0098**^∗∗^	**0**.**0882**
		U	48	2.3388	**0**.**0193**^∗^	**0**.**0869**
	B vs. U	D	48	-1.6384	0.1013	0.2280
		B	48	-0.5800	0.5619	1.0115
		U	48	2.2046	**0**.**0275**^∗^	**0**.**0825**
GU (T1)	B vs. G	D	48	-2.2059	**0**.**0274**^∗^	**0**.**1233**
		B	48	0.5396	0.5895	0.6632
		G	48	1.9856	**0**.**0471**^∗^	**0**.**1413**
	G vs. U	D	48	-1.6121	0.1069	0.2406
		G	48	2.6163	**0**.**0089**^∗∗^	**0**.**0801**
		U	48	-1.3811	0.1673	0.3011
	B vs. U	D	48	-0.7987	0.4244	0.6367
		B	48	0.6486	0.5166	0.6642
		U	48	0.0941	0.9250	0.9250
UG (T2)	B vs. G	D	48	0.4140	0.6789	0.7637
		B	48	2.4044	**0**.**0162**^∗^	**0**.**0729**
		G	48	-2.9378	**0**.**0033**^∗∗^	**0**.**0297**^∗^
	G vs. U	D	48	0.9599	0.3371	0.5056
		G	48	-2.3196	**0**.**0204**^∗^	**0**.**0612**
		U	48	1.9675	**0**.**0491**^∗^	**0**.**1105**
	B vs. U	D	48	0.3041	0.7611	0.7611
		B	48	-1.0113	0.3119	0.5613
		U	48	0.5485	0.5833	0.7500


*^∗^*p* < 0.05, ^∗∗^*p* < 0.01, ^∗∗∗^*p* < 0.001.*

### Differential Conditioning Produces Aversive but Not Safety Learning

In aversive differential conditioning, avoiding the CS+ or seeking the CS- are entangled behaviors, but distinct learning events. In order to characterize more precisely the association(s) formed by the honey bees during our training protocol, we extended the test phase: in addition to testing the CS+ against the CS- as before, we also tested either one separately against a novel stimulus (i.e., CS+ vs. New or CS- vs. New). In the case of odors, we kept the background lights in two of the arms but only presented one of the odors at a time (i.e., CS+ vs. None or CS- vs. None). With this experimental design, a pre-existing bias for or against a stimulus would influence the results. Therefore, we statistically tested the trained bees against an independent group of control bees. Bees in the control group experienced both the CSs and the shocks but not in close temporal association (unpaired control, see Figure 2). We used four training trials for each CS.

**FIGURE 8:**
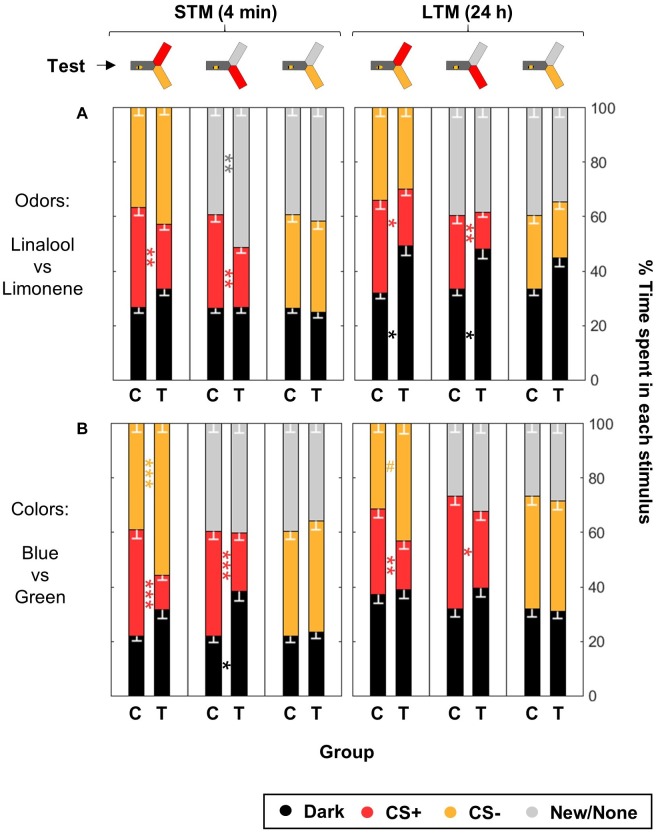
Differential conditioning produces aversive learning of the CS+ but not safety learning of the CS–. During training, shocks were paired with the CS+ but not the CS– for trained animals (T), while control bees (C) received shocks unpaired from both CS. After training, the bees were confronted to three tests: CS+ vs. CS–, CS+ vs. New/None and CS– vs. New/None. Each bee participated in all tests. For each group of bees, the percentages of time spent in the environments available during a given test (e.g., dark, CS+, and CS–) are plotted and stacked into a single bar. Since we included all environments, the resulting stack always totalize 100% (the whole test duration). Data is represented as mean – SEM. *n* = 96 trained bees and 48 control bees for each sensory modality. Mann–Whitney *U*-tests comparing C to T, corrected with FDR, ^∗^*p* < 0.05, ^∗∗^*p* < 0.01, ^∗∗∗^*p* < 0.001, #0.05 > *p* > corrected threshold. **(A)** In the case of odors, bees were trained with Linalool and Limonene, and each odor was tested against a blank control (because of the dim blue illumination used to make the choice arms attractive during odor tests, the choice was thus blue vs. blue + odor). **(B)** In the case of colors, bees were trained with blue and green, and the novel color was UV.

Bees trained for the odorants linalool vs. limonene avoided the CS+ whenever present, but did not change their behavior toward the CS- (Figure 8A and Table 5). This indicates that the CS+ odor had become aversive, but the CS- odor had not changed valence. As in the previous data set, during the LTM test bees spent a larger amount of time in the dark. They did so significantly more than the control bees and only when the CS+ was present. Hence, rather than a general decrease in phototactic behavior or a loss of specificity of the memory, the data indicate that the aversive memory was strengthened while being consolidated during the 24 h between the tests, resulting in stronger aversion of the CS+ that kept the bee away from both choice arms.

**Table 5:** Summary of statistical results for the experiments testing for safety learning (Figure 8), Wilcoxon signed-rank tests, “*p* FDR” indicates the *p*-value corrected with FDR.

Group	Test	Color	*n*_t_ = 2*n*_c_	STM			LTM		
					
				*z*	*p*	*p* FDR	*z*	*p*	*p* FDR
Odors	CS+ vs. CS-	D	96	-1.9390	0.0525	0.0945	-2.8685	**0**.**0041**^∗∗^	**0**.**0123**^∗^
		CS+	96	3.2759	**0**.**0011**^∗∗^	**0**.**0099**^∗∗^	2.9959	**0**.**0027**^∗∗^	**0**.**01215**^∗^
		CS-	96	-1.7128	0.0867	0.1301	1.0460	0.2956	0.3801
	CS+ vs. None	D	96	0.5221	0.6016	0.7735	-2.7182	**0**.**0066**^∗∗^	**0**.**01485**^∗^
		CS+	96	3.1998	**0**.**0014**^∗∗^	**0**.**0063**^∗∗^	3.2526	**0**.**0011**^∗∗^	**0**.**0099**^∗∗^
		None	96	-3.1236	**0**.**0018**^∗∗^	**0**.**0054**^∗∗^	0.2229	0.8236	0.8236
	CS- vs. None	D	96	0.8403	0.4008	0.9018	-1.8974	0.0578	0.1040
		CS-	96	0.3463	0.7291	0.7291	1.8407	0.0657	0.0986
		None	96	-0.5172	0.6050	0.6806	1.0172	0.3090	0.3476
Colors	CS+ vs. CS-	D	96	-1.3807	0.1674	0.3013	-0.4691	0.639	0.9585
		CS+	96	6.5451	**<0**.**0001**^∗∗∗^	**<0**.**001**^∗∗∗^	3.6267	**0**.**0003**^∗∗∗^	**0**.**0027**^∗∗^
		CS-	96	-3.7234	**0**.**0002**^∗∗∗^	**0**.**0006**^∗∗∗^	-2.1341	**0**.**0328**^∗^	**0**.**0984**
	CS+ vs. New	D	96	-2.7046	**0**.**0068**^∗∗^	**0**.**0153**^∗^	-1.9422	0.0521	0.1172
		CS+	96	4.6076	**<0**.**0001**^∗∗∗^	**<0**.**001**^∗∗∗^	3.0392	**0**.**0024**^∗∗^	**0**.**0108**^∗^
		New	96	0.0485	0.9613	0.9613	-1.0516	0.293	0.5274
	CS- vs. New	D	96	-0.4702	0.6382	0.8205	-0.0234	0.9813	0.9813
		CS-	96	-0.4163	0.6772	0.7619	0.18	0.8571	1.1020
		New	96	0.912	0.3618	0.5427	-0.0788	0.9372	1.0544


*^∗^*p* < 0.05, ^∗∗^*p* < 0.01, ^∗∗∗^*p* < 0.001.*

For the visual paradigm, we focused on the blue/green pair for conditioning as it produced the most reliable performance (see Figure 6), and hence UV was the novel stimulus. Nonetheless, the short-term outcome was the same with all the color pairs (as can be seen by looking in details at Figure 7). When tested to choose between the CS+ and the CS- shortly after conditioning, trained bees spent less time than control bees in the CS+, going instead into the arm containing the CS- (Figure 8B and Table 5). This avoidance of the CS+ was also evident when it was presented against the novel stimulus, whereas the behavior toward the CS- did not change, indicating that no association had been formed with the CS-. Similar results were obtained when the bees were tested again 24 h later, indicating that the aversive memory formed was stable for this length of time. Overall, we conclude that in our apparatus, aversive differential conditioning relied exclusively on the bees learning to avoid the shocked CS, and not on safety learning of the CS that was never shocked.

### Trained Bees Spontaneously Escape From the CS+ When Provided With an Alternative

**FIGURE 9:**
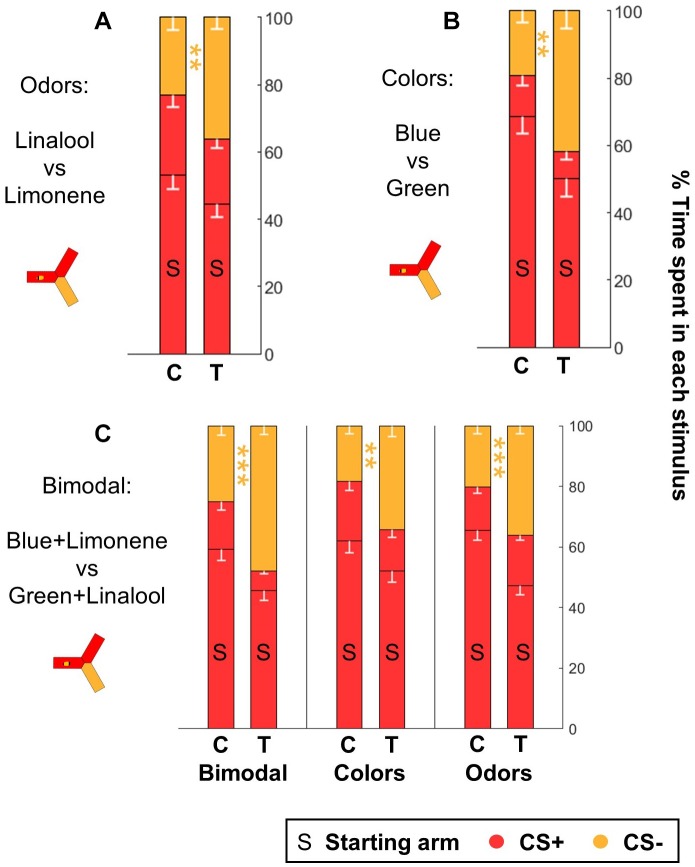
Honeybees learn both color and odor information when trained with bimodal stimuli. After classical training, honeybees spontaneously escaped from the CS+ when they were provided with the CS– as alternative, both after odor **(A)** and after color **(B)** learning (*n* = 48 trained bees and 48 control bees for each sensory modality). **(C)** Honeybees trained in a bimodal task (*n* = 96 trained bees and 96 control bees) performed well both when presented with the same compound stimuli (color+odor), with only the colors or with only the odors. Each bee participated in all three tests after training. Data is represented as mean – SEM. Mann–Whitney *U*-tests comparing C to T, corrected with FDR, ^∗∗^*p* < 0.01, ^∗∗∗^*p* < 0.001.

In the experiments presented above, a choice behavior was induced by presenting two stimuli in two alternative arms, while the bee was in the third, dark arm. These tests took advantage of the positive phototactic behavior exhibited by honey bees inside the yAPIS. If the CS+ induced learned aversion, we figured that a bee should also try to escape from it. We tested this by delivering the CS+ in two arms, including the one occupied by the bee at the start of the test. The third arm always contained the CS-. As before, no shocks were given during the tests, and during the training the US (and the associated CS+) were always delivered in all arms at the same time. Thus, escape behavior was not a useful strategy during the training trials. Nonetheless, we found that trained bees encountering this new test configuration successfully escaped the CS+ arm and stayed in the CS- arm instead, thus spending a higher percentage of their time there than the control bees (Figure 9A,B and Table 6). This was true whether the CSs were colors or odors, thus indicating that honey bees spontaneously tried to escape when presented with the CS+. Interestingly, this protocol provided a way of measuring odor learning without the need for creating phototactic attraction with background illumination.

**Table 6:** Summary of statistical results for the experiments with forced choice tests (Figure 9), Wilcoxon signed-rank tests, “*p* FDR” indicates the *p*-value corrected with FDR.

Group	Test	*n*_t_ = *n*_c_	STM		
			
			*z*	*p*	*p* FDR
Odors	/	48	2.9014	**0**.**0037**^∗∗^	/
Colors	/	48	3.2001	**0**.**0014**^∗∗^	/
Bimodal	Bimodal	96	4.8234	**<0**.**0001**^∗∗∗^	**<0**.**001**^∗∗∗^
	Only colors	96	3.0999	**0**.**0019**^∗∗^	**0**.**0019**^∗∗^
	Only odors	96	4.4974	**<0**.**0001**^∗∗∗^	**<0**.**001**^∗∗∗^


*^∗^*p* < 0.05, ^∗∗^*p* < 0.01, ^∗∗∗^*p* < 0.001.*

### Honey Bees Learn Both Components ina Bimodal Stimulus

Finally, we investigated if the yAPIS could be a good instrument for the study of bimodal learning. We trained honey bees in a simple bimodal task, where the CS was a combination of an odorant and a color stimulus. We used blue + limonene and green + linalool as CSs. As before, the experiments were balanced: half of the bees were trained to associate the shocks with blue+limonene, whereas for the other half the shocks were paired with green + linalool. The performance of the bees was then evaluated by giving a choice between the same bimodal stimuli, or by testing the colors alone or the odors alone (Figure 9C and Table 6). Trained bees spent significantly less time than control bees in the arms that contained the complete CS+ or a component of the CS+. We conclude that the bees had associated both the color and the odor with the shocks, so that each stimulus was sufficient to trigger an aversive response on its own. Thus, the yAPIS provides a robust, lab-based method to further probe how bimodal compounds are integrated and learned.

## Discussion

Animals have to make choices between different environments to escape dangers in many situations, and learn about which environments are dangerous based on previous experience. Choice behaviors can be exploited to study the mechanisms of sensory coding, learning and memory, and decision making (Guerrieri et al., 2005; Hadar and Menzel, 2010; Devaud et al., 2015; Klappenbach et al., 2017). Recent technological developments allow for increasingly automated systems (e.g., Yang et al., 2011; Itskov et al., 2014; Alisch et al., 2018), affording for large-scale screening and standardized conditions.

In this study, we present a new tool for the study of aversive learning with potential to include bimodal forms of training. Walking honey bees are both trained and tested in an automated Y-maze that we named yAPIS, presented here. This system is based on our previous, linear APIS system, where bees could choose between two halves of a linear space environment, that they explored at the beginning (Kirkerud et al., 2013; Wehmann et al., 2015). In the three-arm system presented here, however, bees make repeated left-right choices at a central decision point. We used the yAPIS to perform aversive learning experiments. During the training phase, the apparatus was controlled as a single unit presenting the chosen CS throughout, paired with electric shocks (the US) if appropriate. This arrangement ensured that the association was entirely classical, with no operant components: the animal’s own behavior had no influence on its exposure to the stimuli. Learning was then tested by measuring the place preference of the bee when offered a choice between two alternative stimuli. Using this methodology, we successfully trained bees in olfactory, visual, and bimodal tasks. Learning was already evident after one or two training trials, and four training trials led to a consistent long-term memory after 24 h.

### UV Cannot Be Easily Conditioned in an Aversive Paradigm

We found that not all colors can be learned equally well. Bees did not learn consistently to avoid UV light that was associated with shocks. Bees artificially reared indoor during the winter months performed very erratically when trained with this light. The performance of outdoor summer bees was better, but remained weaker when UV was the CS+ compared to when the shocks were associated with the blue or green lights. The UV light, in absolute terms, was dimmer than the blue or green lights (Figure 1D), thus one concern could be that the bees were not able to perceive this light. However, several observations argue against this explanation. First, in honeybees the short-wavelength receptor is known to be more sensitive than the other two photoreceptors by an order of magnitude (von Helversen, 1972; Lunau and Maier, 1995), thus the photon counts cannot be directly interpreted. Furthermore, our behavioral experiments found that the UV light elicited the same attraction (measured as the time spent in each light) as the blue or green lights in two-choice tests (Figure 1E). Taken together, these observations suggest that our bees were able to see the UV light.

Our learning results are broadly symmetrical to those obtained after appetitive learning: bees trained to associate a colored light with a reward in free-flying experiments do so better and faster within a certain range in the UV, peaking around 420 nm (Menzel, 1967). Naïve bees on their first foraging flight exhibit a preference for the same range of wavelengths, although this innate bias is quickly overridden by appetitive learning (Giurfa et al., 1995). Thus, honey bees seem to be primed to associate a positive valence to UV light, which may explain the low success of our protocol when UV was paired with shocks. Nonetheless this finding is rather surprising given that honey bees are known for the plasticity of their response. In the olfactory system, innate valences of odors, including pheromones, can easily be reversed through training: alarm pheromones can be associated to a reward (Sandoz et al., 2001; Nouvian et al., 2015), while attractive Nasanov compounds can be associated to a punishment (Roussel et al., 2012).

### Bees Do Not Show Safety Learning

During differential conditioning, honey bees are not only exposed to the shocked CS+, but also to a safe CS-. Thus, they could potentially learn which stimulus is associated with the shocks (aversive learning) but also which stimulus signal the absence of shocks (safety learning) (Schleyer et al., 2018). We investigated whether these two forms of learning co-occurred in our protocol by testing the behavior of the bees toward the CS+ and the CS- independently from each other. We found strong evidence for the existence of an aversive memory, but none for one related to the CS-. In a previous work, some indications were found that honey bees exhibited relief learning (Kirkerud et al., 2017). Relief learning is slightly different from safety learning in that the CS- signals the end of the punishment rather than its absence (Gerber et al., 2014). Relief learning is linked to the timing of a stimulus, and proposed neural models for the cellular mechanisms include spike-timing dependent plasticity and bidirectional modulation of the coincidence detection machinery (Gerber et al., 2014). On the other hand, safety learning implies much longer time-scales and the mechanisms supporting this form of learning remain elusive. In vertebrates, the neural substrates supporting these two forms of learning are known to rely on different brain structures (Mohammadi et al., 2014).

### Multi-Sensory Integration During Aversive Training

Honey bees make use of both visual and olfactory information when foraging (Reinhard et al., 2004; Leonard and Masek, 2014), but also when they perform other important tasks such as defending the nest (Nouvian et al., 2016). However, our understanding of multimodal integration remains poor. Early studies postulated that both components of an olfactory-visual compound were learned independently from each other (Couvillon and Bitterman, 1989; Couvillon et al., 1997), a notion that is also supported in our results (Figure 9). Later works, however, found synergistic effects in appetitive training (Gerber and Smith, 1998; Mota et al., 2011). Namely, PER responses to odors were potentiated or refined by a color context. It is important to note that this seemingly asymmetrical role for odors and colors may be the produce of the protocol, since bees always respond more reliably to odors than to colors when harnessed (Avargues-Weber and Mota, 2016), and tend to generalize between the compound stimulus and the odor (Mansur et al., 2018). In our set-up, the behavioral read-out of learning (spatial avoidance of the CS+) was triggered equally well by both colors and odors. Thus, the yAPIS offers the opportunity for more balanced experiments, in which the extent to which visual and olfactory traces interact (or are independent) could be assessed in an aversive context. For example, we could see how trained honeybees would react to ambiguous or contradictory compounds. The mushroom bodies are thought to be responsible for the formation of both appetitive and aversive memory, and studies in *Drosophila* indicate that these two circuits are mostly independent, and act by shifting the balance of common output neurons (Klappenbach et al., 2017; Cognigni et al., 2018). It would be interesting to verify if multi-sensory stimuli are similarly compartmentalized such that the different elements are only integrated at a late stage in the circuitry.

## Data Availability

The raw data supporting the conclusions of this manuscript will be made available by the authors, without undue reservation, to any qualified researcher.

## Ethics Statement

This study was carried out in accordance with the recommendations of the “3Rs” principles as stated in the Directive 2010/63/EU governing animal use within the European Union. The use of (non-transgenic) honey bees for research purposes has been reported to the “Regierungspräsidium” as required.

## Author Contributions

MN and CGG conceived the study. MN designed, performed and analyzed the experiments, and wrote the original draft. MN and CGG edited the manuscript.

## Conflict of Interest Statement

The authors declare that the research was conducted in the absence of any commercial or financial relationships that could be construed as a potential conflict of interest.
