# Supplementary material for: Aversive Training of Honey Bees in an Automated Y-Maze
Source: Front Physiol. 2019 Jun 4;10:678. doi: 10.3389/fphys.2019.00678 (PMC6558987; doi:10.3389/fphys.2019.00678)
Supplement: Supplementary file 1 [file Data_Sheet_1.PDF]

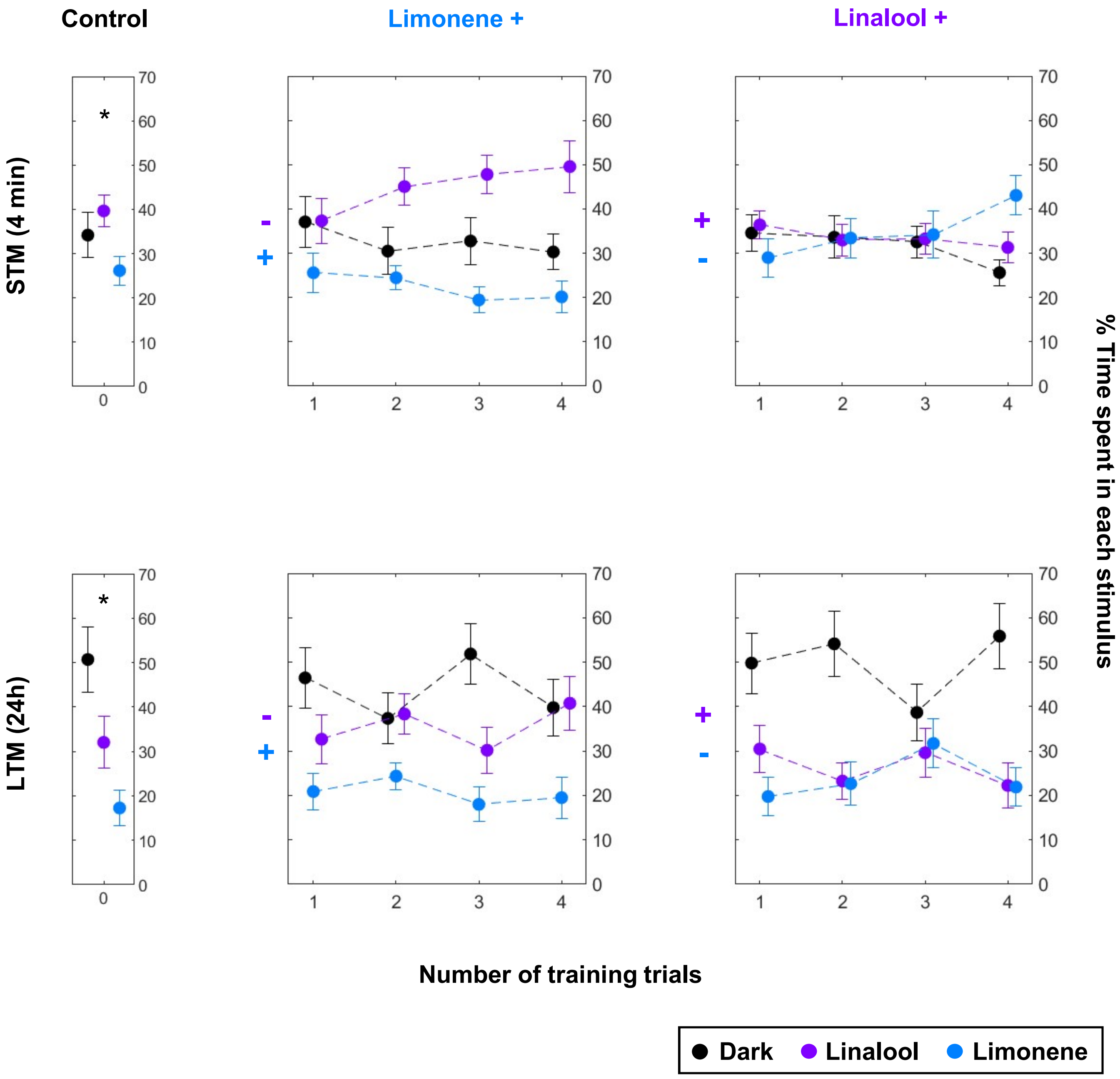

Supplementary figure 1. Details of Fig.5.
During training, shocks were paired with one odor (+) but not with the other (-). The number of training trials was varied from 1 to 4 depending on the group of bees. The same bees were tested twice, 4 min after the end of the training (STM) and again 24 h later (LTM). Data is represented as mean ± s.e.m. Total n = 180 bees (4 numbers of CS presentation x 2 symmetrical trainings x 20 bees + 20 control bees with no training). Bees had an initial preference for linalool over limonene (as seen in the control group with no training, first column - Wilcoxon signed rank tests comparing Linalool to Limonene, * $p<0.05$), thus direct comparisons between the two odors were not performed. This bias was increased when limonene was paired with shocks (second column) and reversed when linalool was paired with shocks (third column).

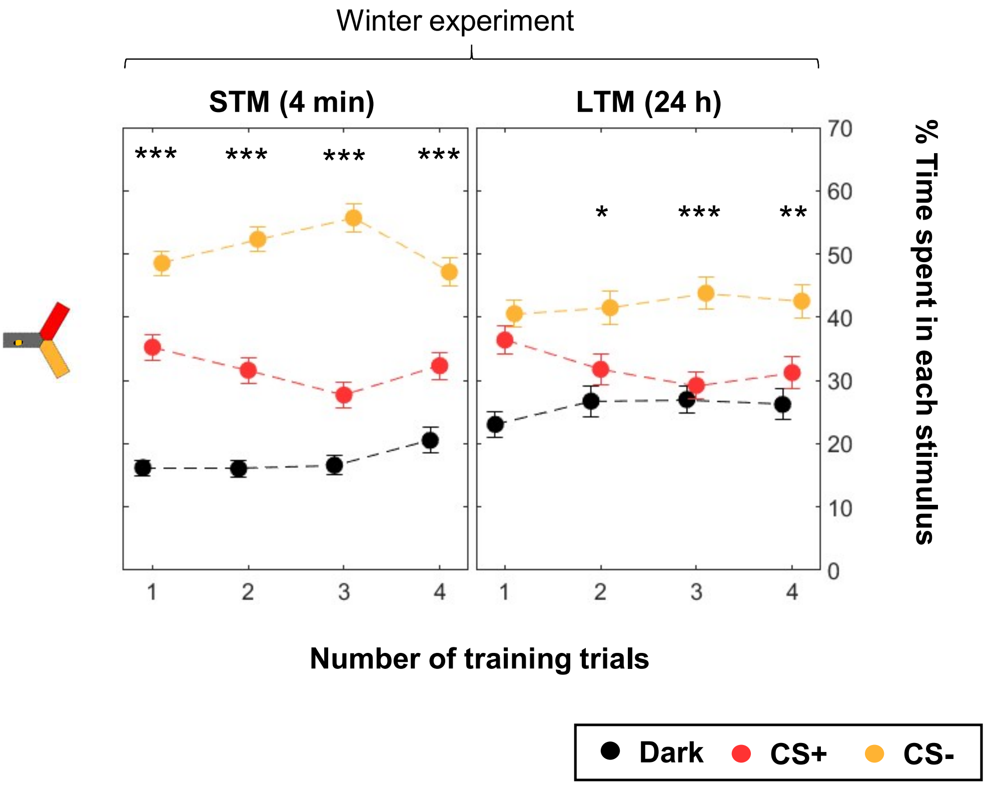

Supplementary figure 2. Differential conditioning of colors.
During training, shocks were paired with the CS+ (red) but not with the CS- (yellow). For each color pair and each number of CS presentations, half of the bees were trained to one color, and the other half to the other color. The same bees were tested twice, 4 min after the end of the training and again 24 h later. Data is represented as mean ± s.e.m. Total n = 480 bees (3 color pairs x 4 numbers of training trials x 40 bees. Wilcoxon signed rank tests comparing CS+ to CS-, * p<0.05, ** p<0.01, *** p<0.001.

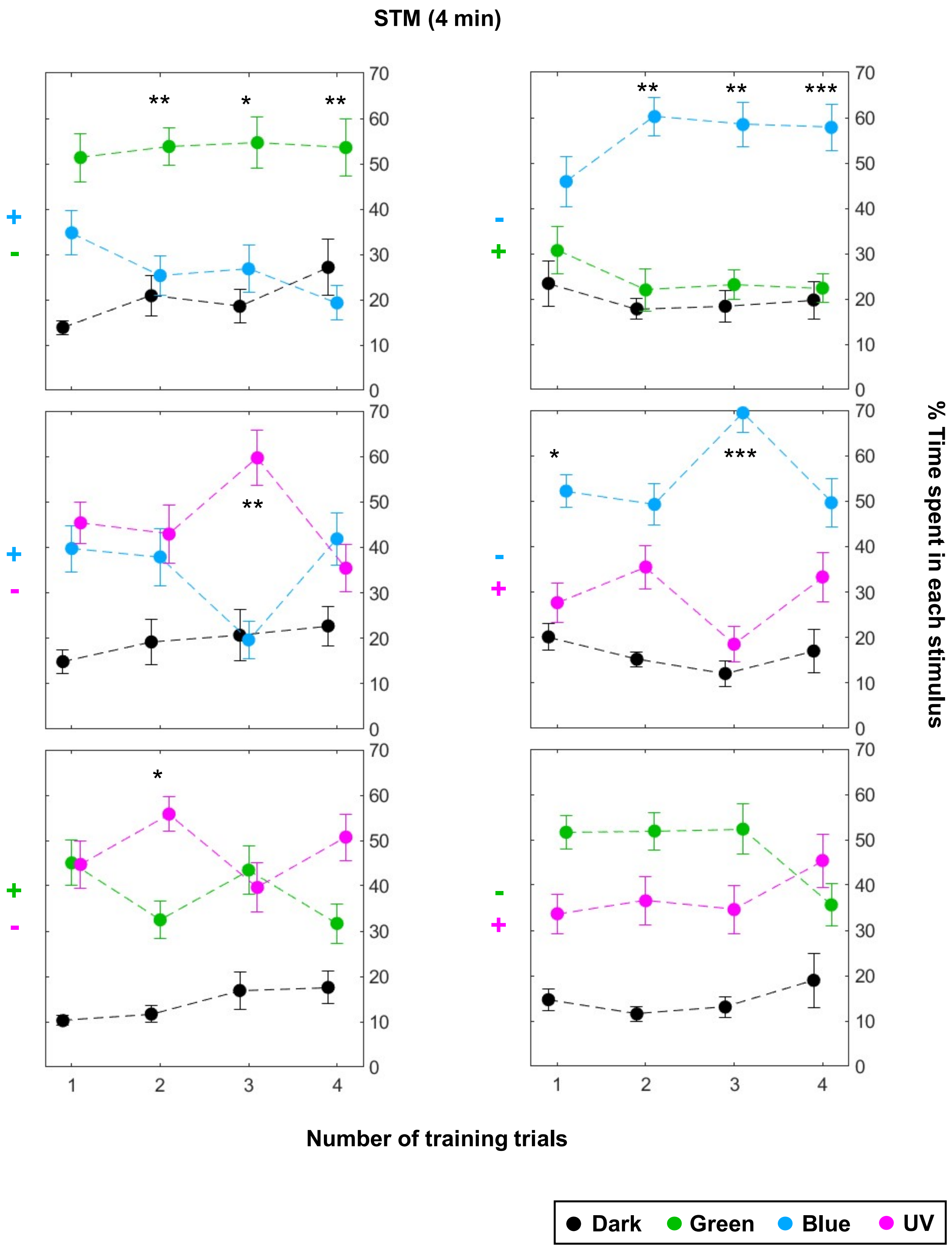

Supplementary figure 3. Details of Fig.6, STM.
During training, shocks were paired with one color (+) but not with the other (-). The number of training trials was varied from 1 to 4 depending on the group of bees. The bees were tested 4 min after the end of the training (STM). Data is represented as mean ± s.e.m. Total n = 480 bees (3 colour pairs x 4 numbers of training trials x 2 symmetrical trainings x 20 bees). Wilcoxon signed rank tests comparing CS+ to CS-, * $p<0.05$, ** $p<0.01$, *** $p<0.001$.

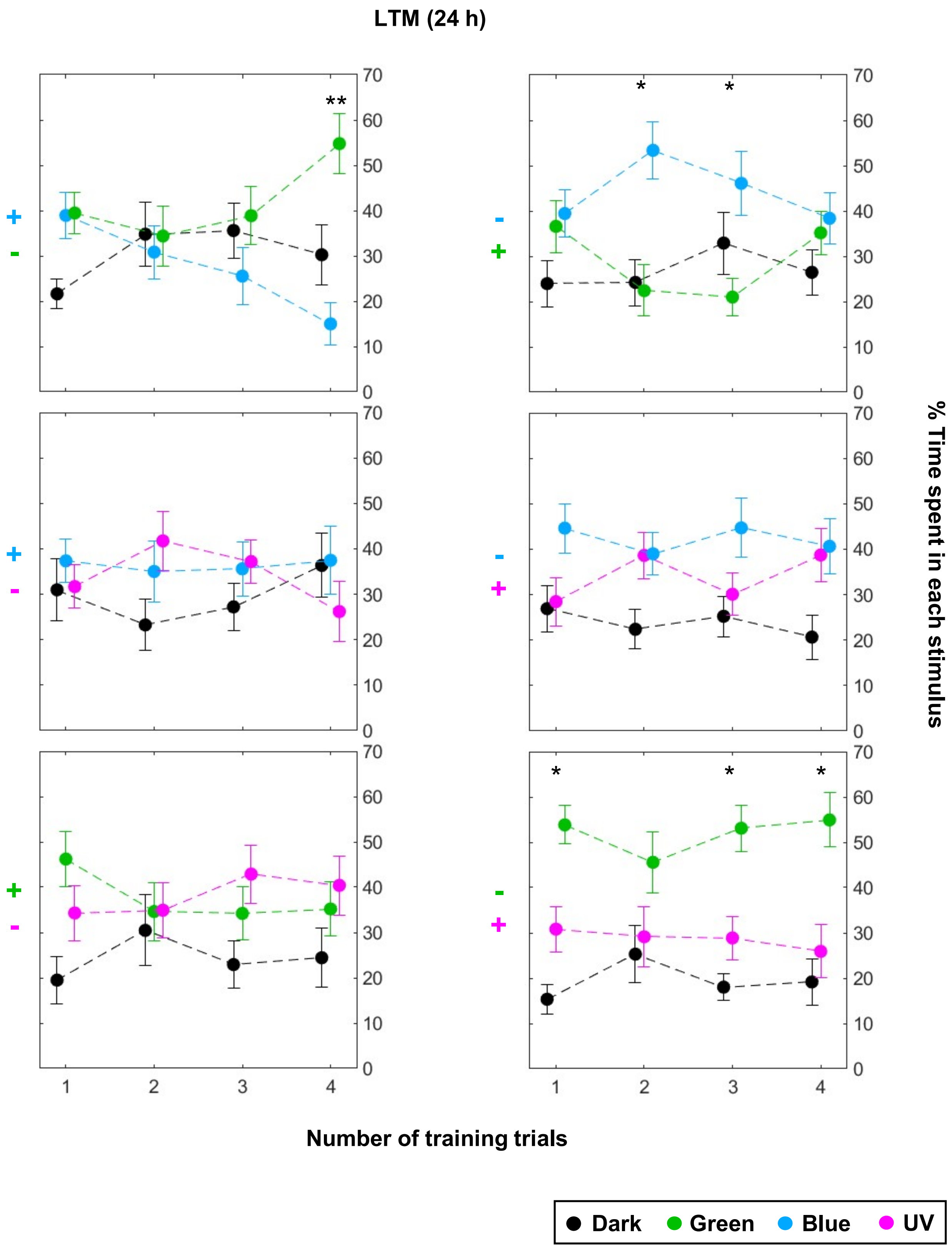

Supplementary figure 4. Details of Fig.6, LTM.

During training, shocks were paired with one color (+) but not with the other (-). The number of training trials was varied from 1 to 4 depending on the group of bees. The bees were tested for the second time, 24h after the training (LTM). Data is represented as mean ± s.e.m. Total n = 480 bees (3 colour pairs x 4 numbers of CS presentation x 2 symmetrical trainings x 20 bees). Wilcoxon signed rank tests comparing CS+ to CS-, * $p<0.05$, ** $p<0.01$.
